# Met-Flow analyses of the metabolic heterogeneity associated with different stages of cord blood-derived hematopoietic cell differentiation

**DOI:** 10.3389/fimmu.2024.1425585

**Published:** 2024-10-17

**Authors:** Sen Zhang, Xiaodong Kong, Ming Yao, Jinfeng Qi, Ying Li, Haoyue Liang, Yuan Zhou

**Affiliations:** ^1^ State Key Laboratory of Experimental Hematology, National Clinical Research Center for Blood Diseases, Haihe Laboratory of Cell Ecosystem, Institute of Hematology & Blood Diseases Hospital, Chinese Academy of Medical Sciences & Peking Union Medical College, Tianjin, China; ^2^ Department of Pharmacology & Regenerative Medicine, University of Illinois Chicago, Chicago, IL, United States; ^3^ Department of Geriatrics, Tianjin Geriatrics Institute, Tianjin Medical University General Hospital, Tianjin, China; ^4^ Tianjin Institutes of Health Science, Tianjin, China

**Keywords:** hematopoietic cells, umbilical cord blood, lineage differentiation, metabolic chart, Met-Flow

## Abstract

**Background:**

The differentiation of hematopoietic cells is significantly affected by cell metabolic activity. However, despite increasing interest in this field, there has been no comprehensive investigation of the metabolic functions of human hematopoietic cells during specific phases of differentiation. Thus, this study was conducted to develop a method for comparing hematopoietic cell lineage differentiation based on the metabolic functions of the cell. The metabolic activity of human umbilical cord-derived hematopoietic cells was examined during various phases of differentiation, specifically, hematopoietic stem cells (HSCs), hematopoietic progenitor cells, and differentiated blood cells. This approach was used to develop comprehensive metabolic maps corresponding to the different stages.

**Results:**

HSCs were found to have robust fatty acid (FA) synthesis, FA oxidation, pentose phosphate pathway (PPP) activity, and glucose uptake, shown by their significantly higher expression of ACAC, CPT1A, G6PD, and GLUT1 as compared to differentiated pluripotent progenitor cells, common myeloid progenitors, megakaryocyte erythroid progenitors, lympho-myeloid primed progenitors, and granulocyte-macrophage progenitor cell populations. In monocytic differentiation, higher levels of ACAC, ASS1, ATP5A, CPT1A, G6PD, GLUT1, IDH2, PRDX2, and HK1 protein expression were evident in classical and intermediate monocytes relative to non-classical monocytes, consistent with high anabolic and catabolic levels. Compared with myelocytes and mature cells, the meta-myelocyte and pro-myelocyte populations of granulocytes show significantly elevated levels of ACAC, ASS1, ATP5A, CPT1A, G6PD, IDH2, PRDX2, and HK. In contrast to naïve and regulatory B cells, pro-B cells had higher levels of oxidative phosphorylation, while regulatory B cells showed greater PPP activity, glucose uptake, and tricarboxylic acid cycle activity. The analyses of T cells also indicated significantly higher ACAC, ASS1, ATP5A, CPT1A, G6PD, GLUT1, IDH2, PRDX2, and HK1 expression levels in CD4+ populations compared with CD8+ populations.

**Conclusions:**

The results provide comprehensive analytical methods and reference values for future systematic studies into the metabolic functions of various cord blood-derived hematopoietic cell populations in different pathological or physiological conditions. These findings could also contribute to research on the connection between cellular metabolism and cancer or aging.

## Introduction

Hematopoietic stem and progenitor cells (HSPCs) are essential for the maintenance of the blood system and immunological homeostasis throughout the human lifespan (1). Hematopoietic stem cell (HSC) transplantation is an effective therapeutic approach to treat specific malignancies and hematological disorders (2); however, the efficacy of this procedure is significantly affected by the quantity and quality of HSCs. The regulation of the maintenance and functionality of these vital progenitor cells is particularly dependent on metabolic activity, which can be influenced by both intrinsic and extrinsic factors (3–6). Mutations in crucial metabolic enzymes may also lead to the malignant transformation of HSCs (7–9). Despite a growing focus on the metabolic functions of these cells in research, most studies to date have only examined the metabolic functions of specific types of hematopoietic cells. Therefore, there is an urgent need for comprehensive analyses of the metabolic functions of cord blood (CB)-derived hematopoietic cells at different phases of differentiation (10–14).

In the mammalian hematopoietic system, HSCs are pluripotent cells that initially undergo differentiation to yield pluripotent progenitor cells (MPPs), which, in turn, differentiate to produce hematopoietic progenitor cells (HPCs) (15–18). These cells have the potential to form common myeloid progenitors (CMPs) and lympho-myeloid primed progenitors (LMPPs), which in turn develop into erythroid, myeloid, and megakaryocytic populations. Alternatively, HPCs can differentiate into granulocyte-macrophage progenitors (GMPs) and megakaryocyte erythroid progenitors (MEPs), which form granulocytes/monocytes and megakaryocytes/erythrocytes, respectively. Granulocytes consist of the pro-myelocyte, myelocyte, meta-myelocyte, and mature stages, whereas monocytes are often classified into the classical, non-classical, and intermediate subtypes. Multipotent lymphoid progenitors (MLPs) and GMPs can be formed from LMPPs via further differentiation (19). Moreover, MLPs can differentiate into B cell/natural killer (B-NK) progenitors and T cells. The former produce mature B and NK cells. The differentiation of B cells is also a multi-stage process that proceeds from the pro-B cell stage to form naive and regulatory B cells. T cells broadly differentiate into the CD4+ and CD8+ T cell subsets. All of these cell populations demonstrate distinct, highly specific functions within the human body.

Previous studies have been unsuccessful in conducting comprehensive analyses of the metabolic functions in different populations of CB-derived hematopoietic cell populations under similar experimental conditions and systemic standards. To compare the metabolic functions of various cells, a Met-Flow multi-parameter flow cytometry approach was implemented to identify 9 critical proteins for analysis. These proteins correspond to key or rate-limiting enzymes that are associated with corresponding metabolic pathways of interest (20). These targets included ACAC and ASS1, which are respectively correlated with fatty acid (FA) synthesis and arginine metabolism (21, 22), as well as HK1, G6PD, IDH2, ATP5A, CPT1A, and GLUT1, involved in catabolic activities and associated with glycolysis, the pentose phosphate pathway (PPP), the tricarboxylic acid (TCA) cycle, oxidative phosphorylation, FA oxidation, and glucose uptake. Moreover, the levels of PRDX2, a protein involved in phosphate metabolism and antioxidant functions, were also analyzed (7, 23, 24). This study aimed to evaluate the metabolic proteins in CB-derived hematopoietic cells during the various phases of differentiation using standardized experimental conditions and protocols to facilitate the comparison of the metabolic features of the various cell lineages. Collectively, the findings will provide a valuable resource that includes basic protocols and reference values that may be employed by others to conduct systematic research on cellular metabolic activity. These results were combined with preliminary transcriptomic analyses to conduct a deeper analysis of the metabolic heterogeneity of hematopoietic cells which will serve as a basis for future research on the connections between cellular metabolic functionality and conditions such as cancer, aging, hematological diseases, and cardiovascular and cerebrovascular diseases.

## Materials and methods

### Human cord blood

Human CB samples were obtained from the Biobank of the Institute of Hematology & Blood Diseases Hospital and Tianjin Cord Blood Bank (Tianjin, China). Mononuclear cells were enriched using HESpan (B.BRAUN, L6511). The study procedure was approved by the Ethics Committee of the Hospital for Blood Diseases of the Chinese Academy of Medical Sciences (KT2020016-EC-2).

### Flow cytometry

Mononuclear cells were incubated with antibodies in PEB solution (PBS with 2% FBS and 2 mM EDTA) for 30 min on ice. For monocyte subtypes (classical, intermediate, non-classical), cells were incubated with an anti-human lineage cocktail (including RPA-2.10, OKT3, 61D3, CB16, HIB19, TULY56, and HIR2) (1:10), anti-human CD45 PE (2D1, 1:100), anti-human CD16 (3G8, 1:100), and anti-human CD14 (63D3, 1:100).

For T cell subtypes (CD4+ and CD8+), cells were treated with antibodies against CD3 (OKT3, 1:100), CD4 (SK3, 1:100) and CD8 (SK1, 1:100). For B cell subtypes (pro-B, naïve B, regulatory B cells), cells were incubated with antibodies against CD19 (HIB19, 1:100), CD24 (ML5, 1:100), CD38 (HB7, 1:100), and CD10 (HI10a, 1:100), while for granulocyte subtypes (pro-myelocyte, myelocyte, meta-myelocyte, and mature), the antibodies used were against CD33 (P67.6, 1:100), CD45 (2D1, 1:100), CD11b (M1/70, 1:100), and CD16(3G8, 1:100).

For hematopoietic stem and progenitor subtypes (HSC, MPP, MLPP, MLP), mononuclear cells were isolated with a CD34 Microbead kit (Miltenyi Biotech, cat. no.130-046-703), and CD34^+^ cells were incubated with anti-human Lineage Cocktail (RPA-2.10, OKT3, 61D3, CB16, HIB19, TULY56, HIR2; 1:10), anti-human CD34 (581; 5:100), anti-human CD38 (HB7, 1:100), anti-human CD45RA (HI100, 1:100), and anti-human CD90 (5E10, 5:100).

All antibodies were fluorescently labeled with a fluorescence minus one (FMO) staining panel. Following incubation with the antibodies, the cells were treated with a secondary donkey anti-rabbit IgG antibody (1:100).

After staining, cells were washed with 3 mL of PEB and then centrifuged (1500 rpm, 5 min). Fixation was then performed by adding 100 μL of Cytofix/Cytoperm to each sample and incubating in the dark for 15 min, followed by the addition of 1 mL of BD Perm Wash buffer. The cells were then centrifuged (2000 rpm, 5 min) and resuspended in 100 μL of BD Cytoperm Permeabilization Buffer Plus for 10 min in the dark on ice. The cells were then washed with 1 mL of BD Perm Wash buffer as above, followed by resuspension in 100 μL of Cytofix & Cytoperm for 5 min at room temperature. After another round of washing using BD Perm Wash buffer, the cells were blocked for 1 h at room temperature using 100 µL of BSA (0.05%). This was followed by the addition of 1 mL of PBE, centrifugation (2000 rpm, 5 min), and the addition of appropriately diluted antibodies specific for 9 key metabolic proteins (1:100 in blocking buffer) for 1 h at room temperature. Cells were then washed with PBE, followed by suspension for 1 h in a solution containing Brilliant Violet 421TM donkey anti-rabbit IgG antibody (1:100) at room temperature. After a final wash with PBE, cells were resuspended in 500 μL of PEB and filtered with 40-70 μm nylon mesh before analysis. Cells were analyzed by FACS Aria III cell sorter (Becton Dickinson, USA).

For the combination of MLP/LMPP/MPP/HSC, a lineage^-^ population was first gated, and based on this, CD34^+^CD38^-^ cell populations were gated, and further MPP (Lin^-^CD34^+^CD38^-^CD45RA^-^CD90^-^), CD90^+^CD45RA^-^ and CD45RA^+^CD90^-^ cell populations were obtained through CD90 and CD45RA gating. HSCs (Lin^-^CD34^+^CD38^-^CD90^+^CD45RA^-^CD49f^+^CD10^-^) were isolated by selecting the CD45RA^-^CD90^+^ cell population via CD49f and CD10. Conversely, MLP (Lin^-^CD34^+^CD38^-^CD90^-^CD45RA^+^CD10^+^) and LMPP (Lin^-^CD34^+^CD38^-^CD90^-^CD45RA^+^CD10^-^) were obtained by selecting the CD90^-^CD45RA^+^ cell population by CD45RA and CD10 (**Supplementray Figure S1A**).

For the combination of B-NK/CMP/GMP/MEP, the lineage^-^ population was gated first, and based on this, the CD34^+^CD38^+^ cell population was gated to obtain B-NK (Lin^-^CD34^+^CD38^+^CD45RA^+^CD10^+^) and CD10^-^ cell populations via selecting CD45RA and CD10 cell population. Next, the CMP (Lin^-^CD34^+^CD38^+^CD10^-^CD135^+^CD45RA^-^), MEP (Lin^-^CD34^+^CD38^+^CD10^-^CD135^-^CD45RA^-^), and GMP (Lin^-^CD34^+^CD38^+^CD10^-^CD135^+^CD45RA^+^) were further obtained through CD45RA and CD135 selection (**Supplementary Figure S1B**). In the combination of classical monocyte/intermediate monocyte/non-classical monocyte, the Lin^-^CD45^+^ cell population was first gated, and then classical monocyte (Lin^-^CD45^+^CD16^-^CD14^+^), intermediate monocyte (Lin^-^CD45^+^CD16^+^CD14^+^), and non-classical monocyte (Lin^-^CD45^+^CD16^+^CD14^-^) were obtained through CD16 and CD14 selection (**Supplementary Figure S1C**).

For the combination of pro-myelocyte/myelocyte/meta-myelocyte/mature neutrophils, the CD33^+^CD45^+^ cell population was gated first, and then pro-myelocytes (CD33^+^CD45^+^CD11b^-^CD16^-^), myelocytes (CD33^+^CD45^+^CD11b^+^CD16^-^), meta-myelocytes (CD33^+^CD45^+^CD11b^+^CD16^low^), and mature neutrophils (CD33^+^CD45^+^CD11b^+^CD16^+^) were obtained via CD16 and CD11b (**Supplementary Figure S1D**). The CD19^+^ cell population was initially gated in the regulatory B/naive B combination, and then regulatory B (CD19^+^CD24^+^CD38^+^) and naive B (CD19^+^CD24^-^CD38^-^) were identified through CD24 and CD38 gating (**Supplementary Figure S1E**). Moreover, Pro-B (CD19^+^CD10^+^CD34^+^) was obtained by selecting CD19^+^ cell populations through the CD10 and CD34 gate (**Supplementary Figure S1F**). In the combination of CD4+T/CD8+T, the CD3^+^ cell population was gated, and based on this, CD4^+^T (CD3^+^CD4^+^CD8^-^) and CD8+T (CD3^+^CD4^-^CD8^+^) are obtained through CD4 and CD8 gating (**Supplementary Figure S1G**).

### Transcriptomic analyses

Datasets containing transcriptomic data specific to hematopoietic cells were accessed and downloaded from the NCBI database (GSE24759). The expression of 9 key metabolic genes was analyzed in these cells. Single-cell transcriptomic data were accessed and downloaded using the NCBI database (GSM3044498_CB_CB35). Cell type annotations were developed corresponding to the marker genes found using Seurat. The FindAll Markers function was implemented to locate signature genes associated with each cell type, and UMAP visualizations were constructed with a n_neighbors setting of 15. Clusters were identified by analyzing the expression of hematopoietic marker genes. The default parameters were used in Seurat to identify differentially expressed genes (DEGs) using the FindMarkers function.

### Data analyses

All flow cytometry analyses were conducted using a BD AriaIII flow cytometer (BD Biosciences, USA). The results were analyzed with FlowJo software v10.6.1 (BD Biosciences) to calculate the mean fluorescence intensity (MFI) values for each channel and to construct respective flow plots.

### Statistical analysis

Data were statistically analyzed using SPSS 26.0. Normally distributed data are expressed as mean ± SD. Differences in categorical data were evaluated by ANOVA. Intergroup comparisons were evaluated using Tamhane’s T2 method with uneven variance and the LSD method with uniform variance. Non-normally distributed data are presented as medians with interquartile ranges, and inter-group differences were examined via non-parametric ANOVA–Kruskal-Wallis tests. *P*-values ≤ 0.05 were considered statistically significant. Graphs were plotted using GraphPad Prism 6 software.

## Results

### Analyses of the metabolic heterogeneity of HSPCs

**Figure 1:**
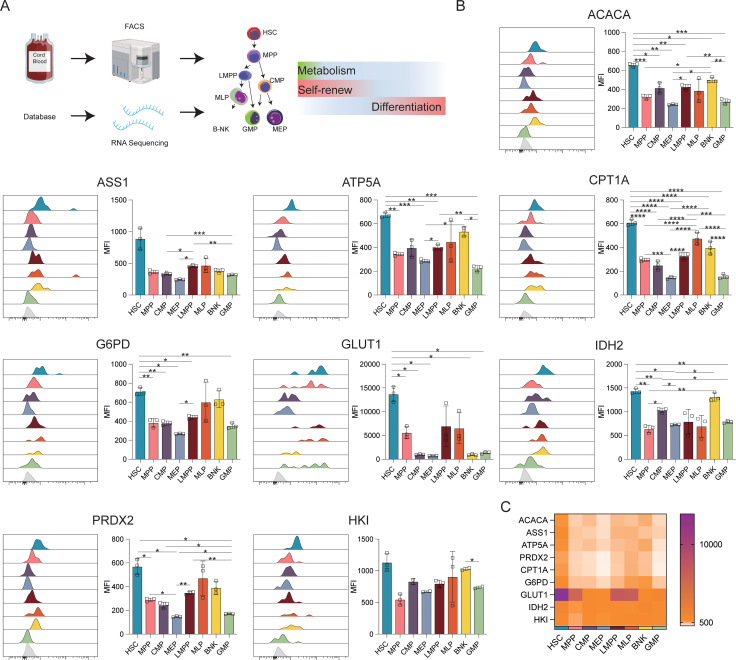
Expression of key metabolic proteins in HSPCs. **(A)** Schematic representation of the study design. The following populations of cord blood cells were evaluated using FACS: HSC, hematopoietic stem cells; MPP, multipotent progenitor; LMPP, lymphoid-primed multipotent progenitor; multi-lymphoid progenitor; B-NK, B-NK cell progenitor; CMP, common myeloid, progenitor; MEP, megakaryocyte-erythroid progenitor; GMP, granulocyte/macrophage progenitor. Data for RNA-seq analysis were obtained from the NCBI database (GSE24759, GSM3044498_CB_CB35 datasets). **(B)** Flow cytometry analysis of the expression of ACACA, ATP5A, CPT1A, G6PD, GLUT1, IDH2, PRDX2, and HK1. **(C)** Heatmap showing the expression of specific proteins in HSC, MPP, CMP, LMPP, MLP, BNK, and GMP cell populations. The light gray color in the histogram indicates the fluorescence minus one (FMO) control. *P ≤ 0.05, **P ≤ 0.01, ***p ≤ 0.001, ****p = 0.000.

To investigate the heterogeneity of cells during the initial stages of human hematopoiesis, CB transcriptomic sequencing data from the NCBI database were analyzed. The profiles of genes associated with anabolic (*ACAC, ASS1*) and catabolic (*HK1, G6PD, IDH2, ATP5A, CPT1A, GLUT1*) activities, and phosphate transport/antioxidant metabolism (*PRDX2*) were evaluated. Flow cytometry was then used to verify differences in the expression of these proteins via flow cytometry (**Figure 1A**).

**Figure 2:**
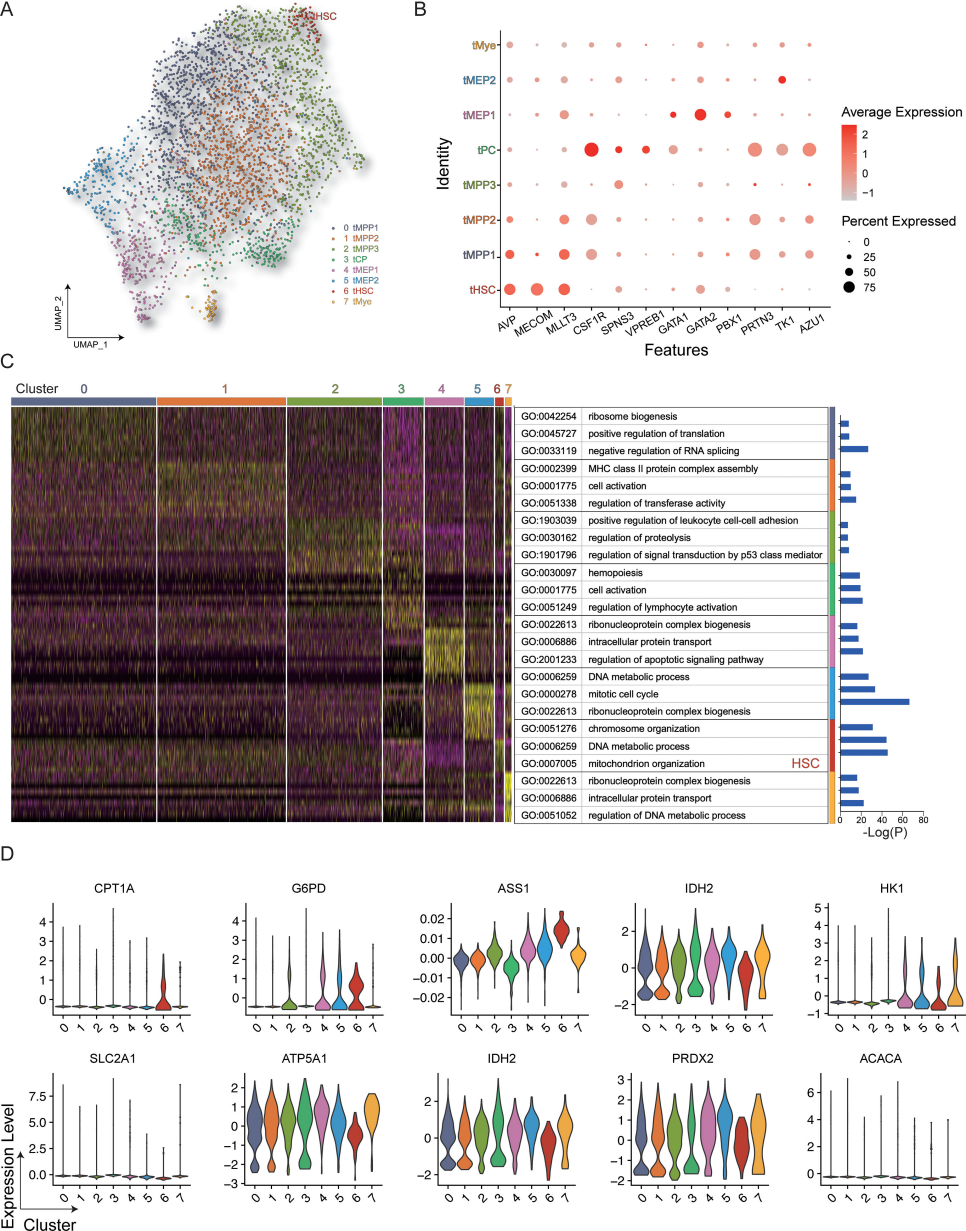
Gene expression in HSPCs. **(A)** All hematopoietic cell clusters (GSM3044498_CB_CB35) were visualized using UMAP plots. **(B)** Dot plot representing the key genes associated with each cluster. **(C)** Heatmap showing differentially expressed genes in the clusters with GO annotations. **(D)** Violin plot of key genes in the different clusters.

The uniform manifold approximation and projection (UMAP) algorithm was employed to evaluate heterogeneity and identify distinct cellular subsets, resulting in the identification of 8 distinct clusters with different cellular identities (**Figures 2A, B**). It can be seen in the UMAP diagram that cells with similar protein expression patterns showed significant clustering, while cell populations with similar phenotypes are seen as highly interconnected node sets, thus enabling the visualization of different cell subpopulations. The final cell classification can be visualized in the form of color dimensions overlaid on UMAP maps or in the form of heatmaps. The proximity of cells in the UMAP maps reflects their distance in high-dimensional space. The genes expressed in each cluster were then annotated using Gene Ontology (GO) (**Figure 2C**), showing that the HSC population expressed high levels of genes associated with mitochondrial organization, which is consistent with distinct metabolic activity. HSCs showed higher expression of both *CPT1A* and *G6PD* compared with the other analyzed cell types (**Figure 2D**).

**Figure 3:**
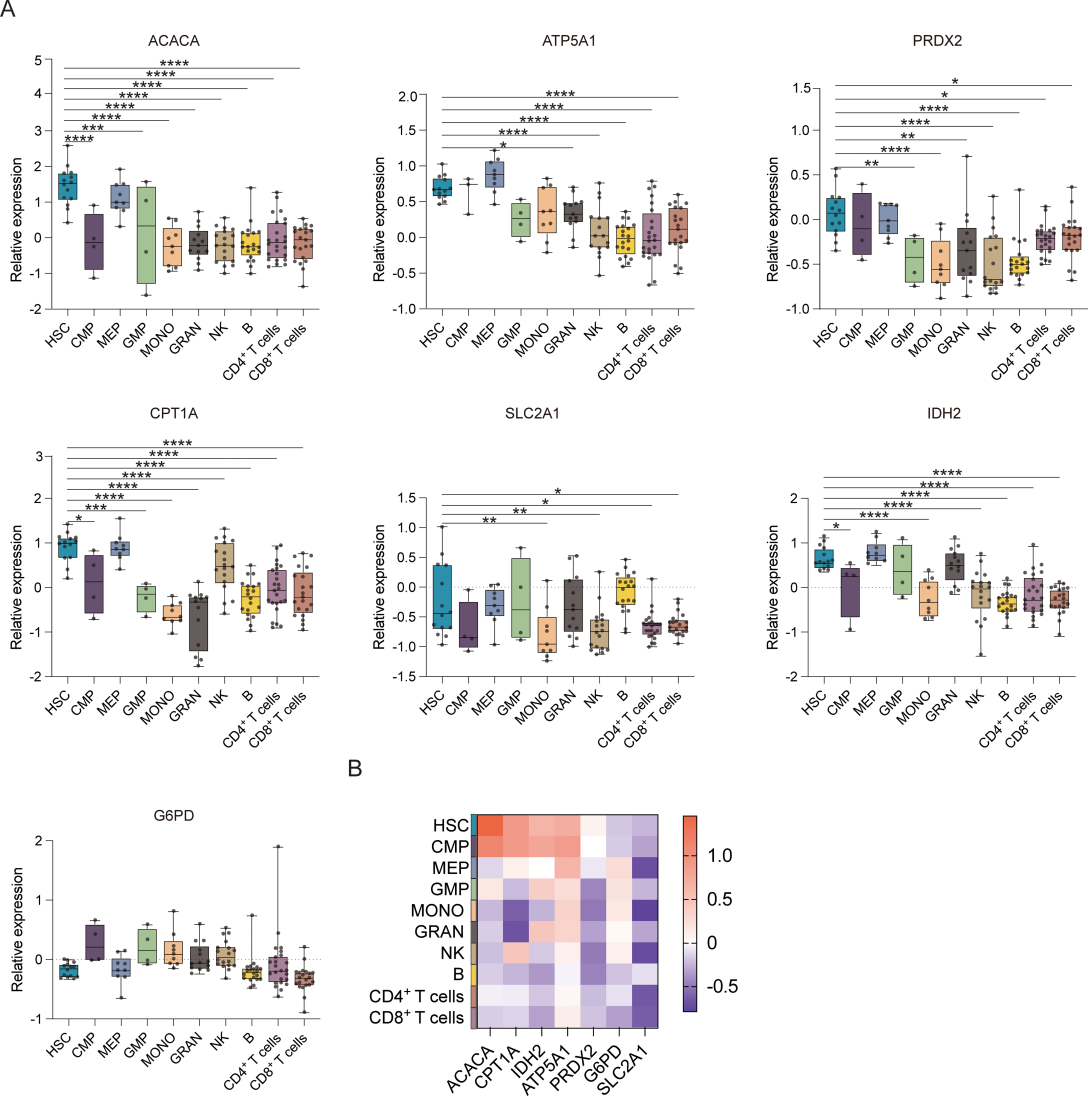
Gene expression profiles of hematopoietic cell populations. **(A)** Boxplots showing the mRNA expression of *ACACA, ATP5A, PRDX2, CPT1A, SLC2A1 (GLUT1), G6PD*, and *IDH2* in the HSC, CMP, MEP, GMP, MONO, GRAN, NK, B, CD4^+^ T, and CD8^+^ T cell populations. **(B)** Heatmap showing the expression of specific genes in the HSC, CMP, MEP, GMP, MONO, GRAN, NK, B, CD4^+^ T, and CD8^+^ T cell populations. *P ≤ 0.05, **P ≤ 0.01, ***p ≤ 0.001, ****p = 0.000.

**Figure 1B** displays the results of protein-level analyses of various key proteins in HSPCs at different stages of differentiation. The results showed significantly higher levels of ACAC, CPT1A, G6PD, and GLUT1 in HSCs relative to the MPP, CMP, MEP, LMPP, and GMP cell populations (*p* ≤ 0.05), consistent with the higher levels of FA synthesis and oxidation, PPP activity, and glucose uptake in HSCs. In comparison to MEPs and GMPs, HSCs also showed markedly higher ATP5A, IDH2, and PRDX2 expression, in correspondence with the increases in oxidative phosphorylation, TCA cycle activity, and phosphate transport metabolism in these cells. The Met-Flow results were consistent with these expression profiles, suggesting that metabolic activity was higher in HSCs compared with other progenitor cell populations (**Figures 1C**, **3**). The transcriptomic analysis indicated higher levels of ACAC, ATP5A, PRDX2, CPT1A, GLUT1, and IDH2 in HSCs relative to those in CMP, MEP, GMP, and other progenitor cells, as well as in mature cells such as monocytes, granulocytes, and lymphocytes (**Figures 3A, B**).

### Analyses of monocyte metabolic heterogeneity

**Figure 4:**
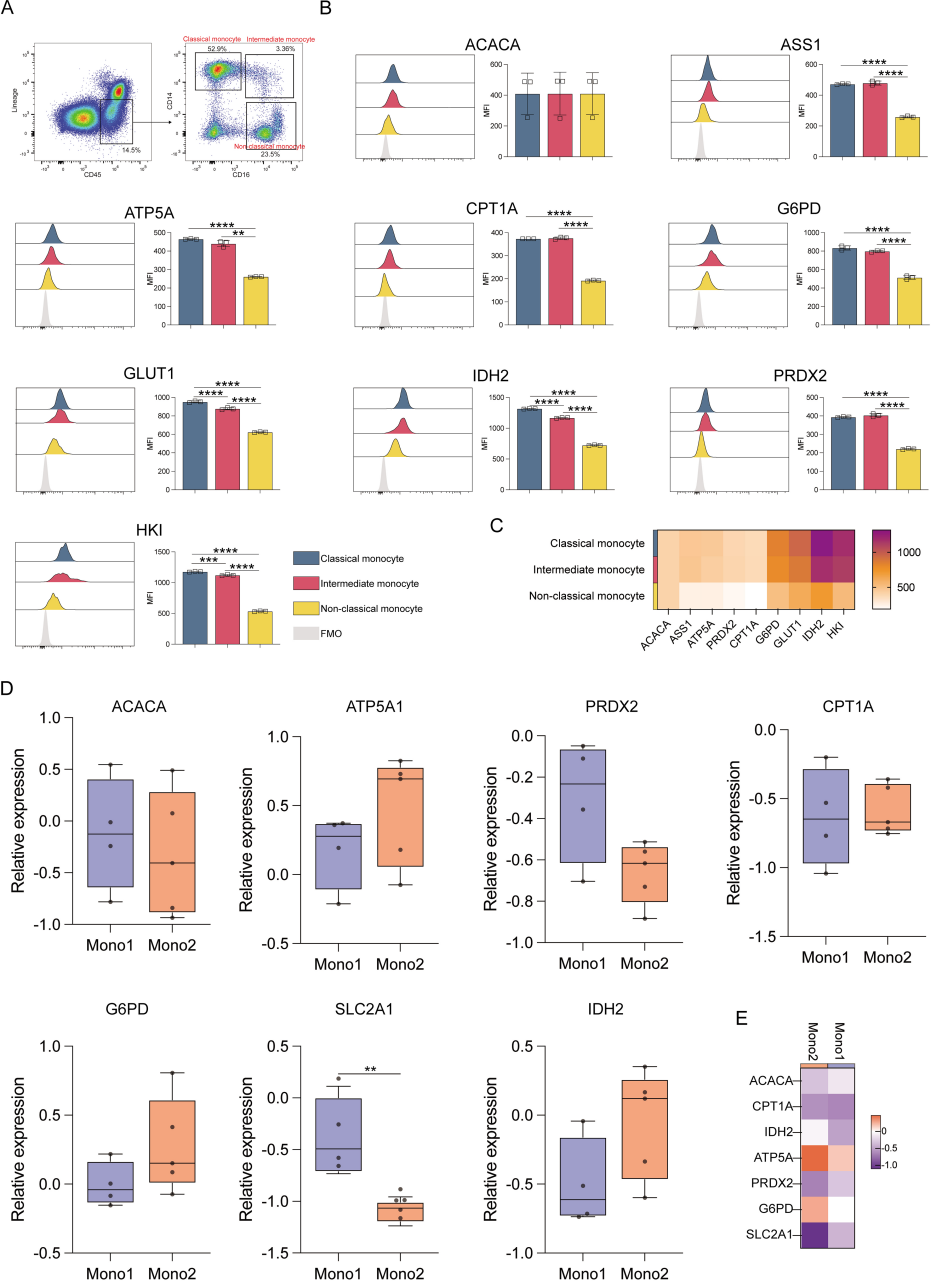
Expression of key metabolic proteins in monocytes. **(A)** Representative flow cytometry plots showing the gating strategy for classical, intermediate, and non-classical monocyte populations. **(B)** Flow cytometry analyses of ACACA, ATP5A, CPT1A, G6PD, GLUT1, IDH2, PRDX2, and HK1 expression. **(C)** Heatmap showing the expression of specific proteins in classical, intermediate, and non-classical monocyte populations. **(D)** Box plot corresponding to the mRNA level expression of *ACACA, ATP5A, PRDX2, CPT1A, SLC2A1 (GLUT1), G6PD*, and *IDH2* in Mono1 (CD34^-^CD33^+^CD13^+^) and Mono2 (FSC^hi^SSC^low^CD14^+^CD45^dim^) populations. **(E)** Heatmap representing the expression of specific genes in Mono1 (CD34^-^CD33^+^CD13^+^) and Mono2 (FSC^hi^SSC^low^CD14^+^CD45^dim^) populations. The light gray color in the histogram indicates the fluorescence minus one (FMO) control. **P ≤ 0.01, ***p ≤ 0.001, ****p = 0.000.

Analysis of different monocyte subsets showed that classical and intermediate monocytes had significantly higher expression of ACAC, ASS1, ATP5A, CPT1A, G6PD, GLUT1, IDH2, PRDX2, and HK1 proteins compared to non-classical monocytes (*p* ≤ 0.01) (**Figure 4A**). This indicates that the former two populations have elevated anabolic and catabolic activities. Classical monocytes also showed higher IDH2 and HK1 levels as compared to intermediate monocytes, consistent with robust glycolysis and TCA cycle activity (**Figures 4B, C**). The results of the Met-Flow analysis were also consistent with their expression profiles (**Figures 4D, E**).

### Analyses of the metabolic heterogeneity of granulocytes

**Figure 5:**
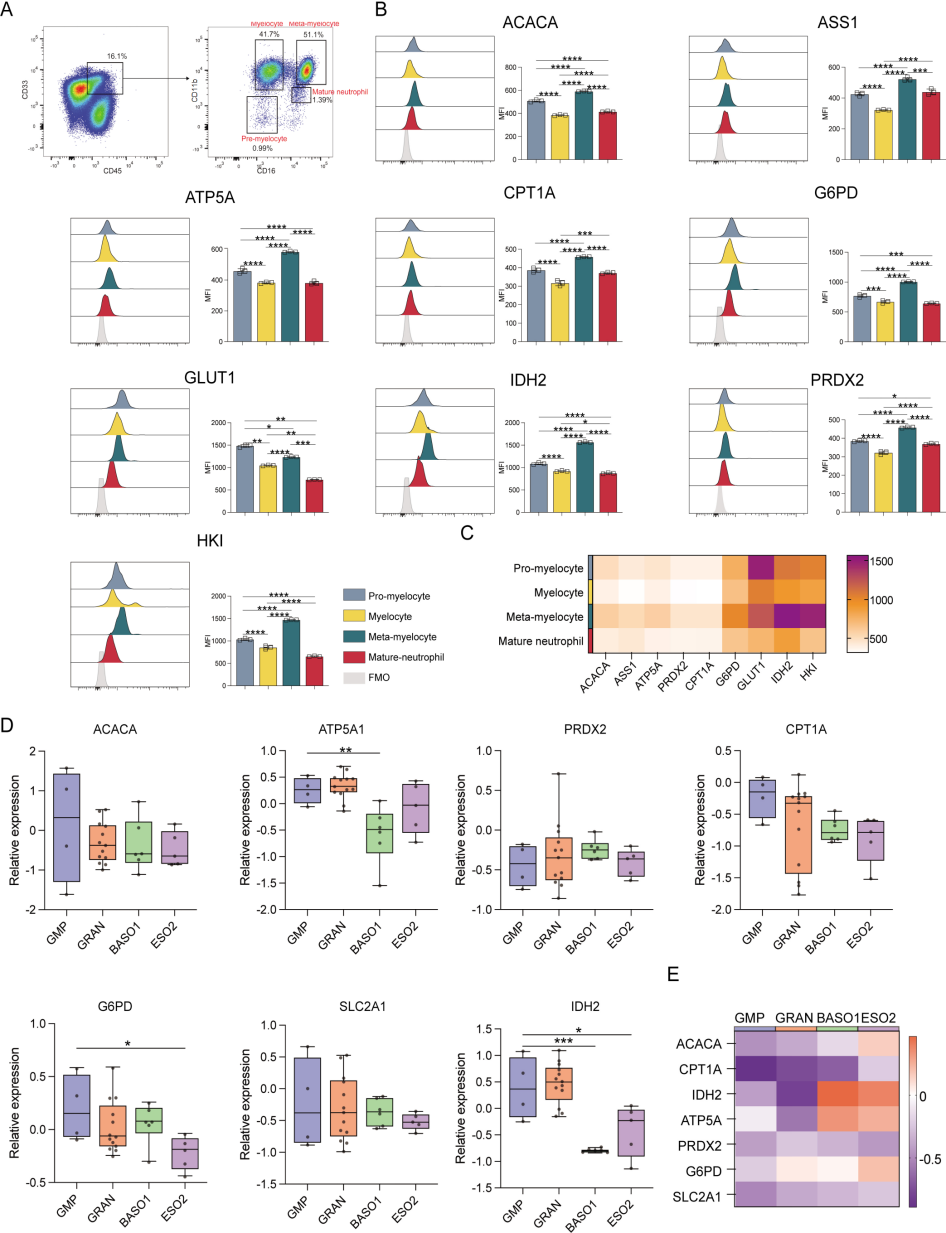
Expression of key metabolic proteins in granulocytes. **(A)** Representative flow cytometry plots showing the gating strategy for pre-myelocyte, myelocyte, meta-myelocyte, and mature neutrophil populations. **(B)** Flow cytometry analyses of the expression of ACACA, ATP5A, CPT1A, G6PD, GLUT1, IDH2, PRDX2, and HK1. **(C)** Heatmap showing the expression of specific proteins in pre-myelocyte, myelocyte, meta-myelocyte, and mature neutrophil populations. **(D)** Box plot depicting the mRNA level expression of *ACACA, ATP5A, PRDX2, CPT1A, SLC2A1 (GLUT1), G6PD*, and *IDH2* in GMP (CD34^+^CD38^+^IL-3Ra^low^CD45RA^+^), GRAN (CD34^-^SSC^hi^CD45^+^CD11b^-/+^CD16^-^/FSC^hi^SSC^low^CD11b^+^CD16^+^), BASO1 (FSC^hi^SSC^low^IL-3Ra^+^CD33^dim+^), and ESO2 (FSC^hi^SSC^low^IL-3Ra^+^CD33^dim+^) populations. **(E)** Heatmap showing with the expression of specific genes in GMP (CD34^+^CD38^+^IL-3Ra^low^CD45RA^+^), GRAN (CD34^-^SSC^hi^CD45^+^CD11b^-/+^CD16^-^/FSC^hi^SSC^low^CD11b^+^CD16^+^), BASO1 (FSC^hi^SSC^low^IL-3Ra^+^CD33^dim+^), and ESO2 (FSC^hi^SSC^low^IL-3Ra^+^CD33^dim+^) populations. The light gray color in the histogram indicates the fluorescence minus one (FMO) control. *P ≤ 0.05, **P ≤ 0.01, ***p ≤ 0.001, ****p = 0.000.

When granulocytes were evaluated (**Figure 5A**), meta-myelocytes were found to have substantially higher levels of ACAC, ASS1, ATP5A, CPT1A, G6PD, IDH2, PRDX2, and HK than pro-myelocytes, myelocytes, and mature neutrophils (*p* ≤ 0.05). This suggests that meta-myelocytes have high catabolic and anabolic activities. A more robust glucose uptake by pro-myelocytes was suggested by their higher GLUT1 levels in comparison to myelocytes, meta-myelocytes, and mature neutrophils (*p* ≤ 0.05) (**Figures 5B, C**). Pro-myelocyte ACAC, ASS1, ATP5A, CPT1A, G6PD, IDH2, PRDX2, and HK expression levels were also higher than those in myelocytes and mature neutrophils (*p* ≤ 0.05), suggesting that the anabolic and catabolic activities of pro-myelocytes were lower to that of meta-myelocytes (**Figures 5D, E**). The transcriptomic data indicated that ATP5A, G6PD, and IDH2 levels in GMPs were higher than those in granulocytes, indicating that GMPs are more active in terms of synthesis and degradation than granulocytes (**Figures 5D, E**).

### Analyses of the metabolic heterogeneity of B cells

**Figure 6:**
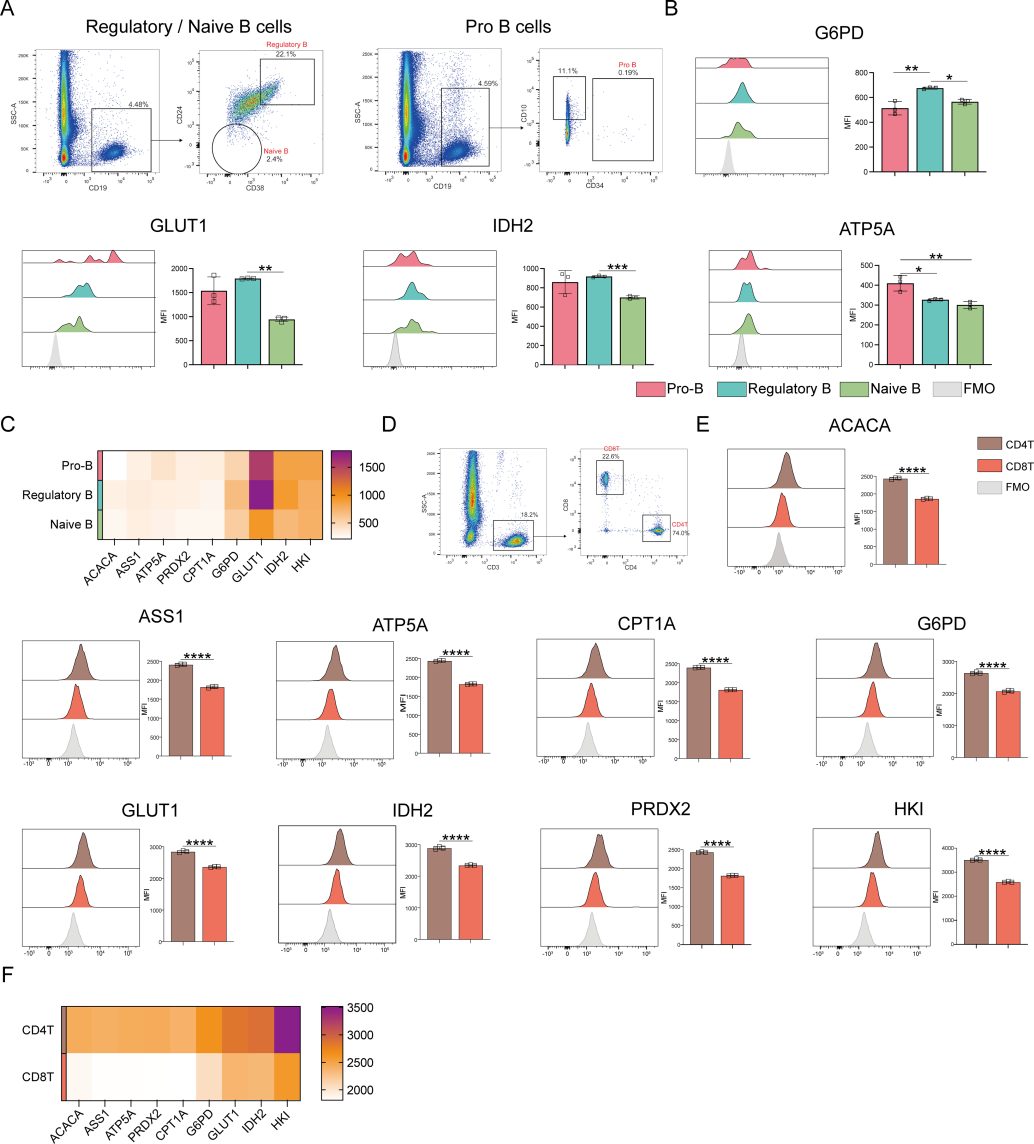
Expression of key metabolic proteins in B and T cells. **(A)** Representative flow cytometry plots showing the gating for pro-B cells, Regulatory B cells, and naive B cells. **(B)** Flow cytometry analyses of ACACA, ATP5A, CPT1A, G6PD, GLUT1, IDH2, PRDX2, and HK1 expression. **(C)** Heatmap showing the expression of key proteins associated with pro-B cells, regulatory B cells, and naïve B cells. **(D)** Representative flow cytometry plot showing the gating for CD4^+^ T and CD8^+^ T cells. **(E)** Flow cytometry analysis of ACACA, ATP5A, CPT1A, G6PD, GLUT1, IDH2, PRDX2, and HK1 expression. **(F)** Heatmap showing the expression of specific proteins in CD4^+^ and CD8^+^ T cells. The light gray color in the histogram indicates the fluorescence minus one (FMO) control. *P ≤ 0.05, **P ≤ 0.01, ***p ≤ 0.001, ****p = 0.000.

ATP5A levels were markedly higher in pro-B cells than in regulatory and naive B cells when comparing the different B-cell populations (*p* ≤ 0.05) (**Figure 6A**). Oxidative phosphorylation was also higher in pro-B cells than in naive and regulatory B cells (**Figures 6B, C**). In contrast to naive B cells, regulatory B cells revealed significantly higher levels of G6PD, GLUT1, and IDH2. This implies that regulatory B cells show enhanced glucose uptake, PPP, and TCA cycle activities.

### Analyses of the metabolic heterogeneity of T cells

**Figure 7:**
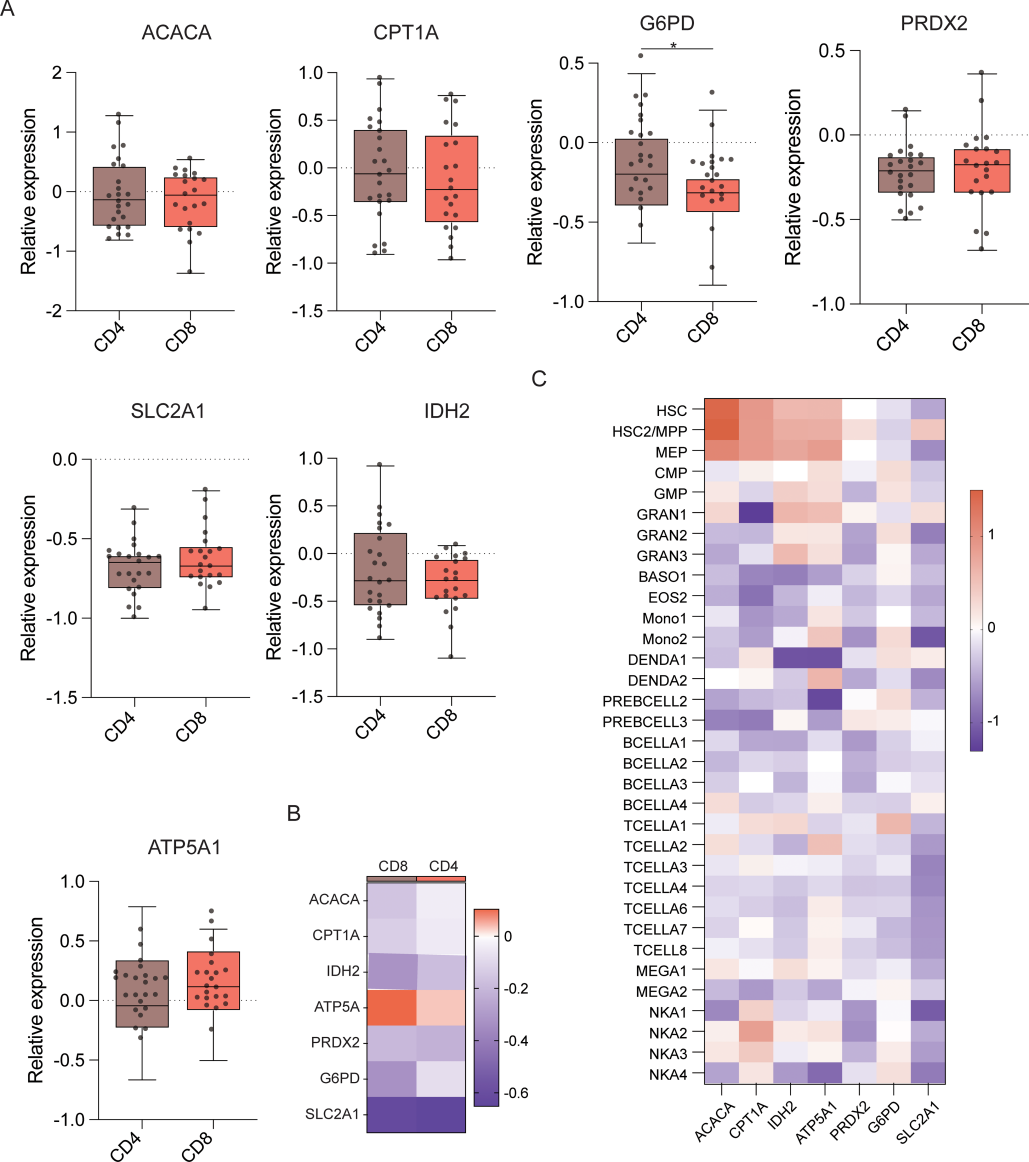
Expression of key metabolic proteins in T cells. **(A)** Box plots showing mRNA expression of *ACACA, ATP5A, PRDX2, CPT1A, SLC2A1 (GLUT1), G6PD*, and *IDH2* in CD4^+^ and CD8^+^ T cells. **(B)** Heatmap showing the expression of specific genes in CD4^+^ and CD8^+^ T cells. **(C)** Heatmap showing the expression of specific genes in all cell populations. *P ≤ 0.05.

Next, T cell subset analyses were performed (**Figure 6D**), which demonstrated that CD4^+^ T cells demonstrated significantly higher levels of ACAC, ASS1, ATP5A, CPT1A, G6PD, GLUT1, IDH2, PRDX2, and HK1 expression than CD8^+^ T cells (**Figures 6E, F**). This indicates that CD4^+^ T cells have better antioxidant, catabolic, and anabolic properties in comparison to CD8^+^ T cells (**Figures 7A–C**). In the transcriptomic analysis, the level of G6PD in CD4+ T cells was significantly higher than that of CD8+ T cell subset (p≤ 0.05), indicating that the metabolic level of the PPP in CD4+ T cell subset is significantly higher than that of CD8+ T cell subset.

## Discussion

The transcriptomic analyses performed in this study suggested that relative to other cell populations, HSCs expressed particularly high levels of ACAC, ATP5A1, PRDX, CPT1A, SLCA1 (GLUT1), and IDH2. In turn, the metabolic functions of human CB-derived hematopoietic cells were compared at crucial stages of differentiation, using similar experimental conditions and standards. These analyses confirmed that HSCs show higher levels of ACAC, CPT1A, G6PD, and GLUT1 expression in comparison to MPP, CMP, MEP, LMPP, and GMP populations. This is in line with the high FA synthesis, FA oxidation, PPP, and glucose uptake activity of these cells.

Met-Flow is a method that combines intracellular staining and flow cytometry to simplify the single-cell analysis of various metabolic pathways of interest. This approach allows the potent examination of the associations between hematopoietic cell subsets and specific pathways (25). This method allows for the assessment of the metabolic potentials of hematopoietic cells by analyzing the protein expression of crucial rate-limiting enzymes related to specific metabolic pathways. Higher expression levels indicate that cells have a greater capacity for using these pathways, as well as greater flexibility in adjusting their metabolic requirements in response to varying nutrient availability and redox conditions. The Met-Flow analyses performed here revealed unique metabolic protein signatures associated with particular subsets of progenitor, granulocyte, monocyte, B, and T cells derived from CB. Compared to more traditional metabolism-focused analytical techniques, Met-Flow is better suited to the simultaneous capture of dynamic metabolic changes in multiple subsets of hematopoietic cells. This strategy can thus provide clearer insight into the metabolic heterogeneity of these different hematopoietic cell subsets in terms of immune metabolism. To establish a basis for the identification of novel metabolic targets that are amenable to therapeutic intervention in a clinical setting, it should be feasible to investigate metabolic reprogramming in various cell populations and their subsets, including CB-derived hematopoietic cells, via Met-Flow-based methods.

The first effective application of CB transplantation was in 1988 to treat a case of Fanconi anemia. Since then, CB has been recognized as an effective alternative source of HSCs for allografting. Therefore, a large number of CB banks have been established globally, and it is now estimated that over 750,000 clinical-grade units of CB exist (26). To simplify CB transplantation, it is crucial to establish standardized, integrated systems (27). Metabolic activity is a crucial factor in the function of HSCs, and the current findings provide valuable insights into the primary metabolic dynamics that define the functionality of CB-derived HSCs.

To ensure the consistency of the experimental results, the same protocol, flow cytometry equipment, and voltage for the target proteins in all staining panels were used throughout the study. To maintain clarity and accuracy, each group in this study was classified according to the name of its corresponding population (28). The analysis of RNA expression was conducted on data from the NCBI database (GSE24759). Transcript levels were processed from data image files using the RMA method using Bioconductor R. To reduce batch effects, transcript levels were further corrected with the ComBat method, which applies an empirical Bayes framework for adjusting data for batch effects. One significant drawback of this study is the inability to conduct Met-Flow and sequencing analyses on CB-derived hematopoietic cells. This is primarily due to the limited availability of instrumentation and antibodies. Future studies can overcome this limitation, allowing the assessment of the metabolic characteristics of human HSPCs, monocytes, granulocytes, T cells, and B cells in more detail, thus providing a strong basis for attempts to improve understanding of metabolic functions in hematopoietic cells.

In this study, the blood of all volunteers was analyzed and the results were verified by experienced pathologists. This ensured that the selected cases were representative. Potential risk factors for various diseases were also investigated in the volunteers by conducting a medical history inquiry, laboratory examination, and rechecking the medical history, and excluding those with diseases, thus reducing potential impacts of factors such as the age and glucose sensitivity of the mother. The CB sources were all of childbearing age, and none were elderly or high-risk. The CB sources also received standardized nutrition and nutritional guidance before delivery. Furthermore, the potential influence of individual differences was reduced by the strict adherence to standardized conditions during CB collection and processing. Moreover, considering the heterogeneity between individuals, the correct use of statistical methods in data analysis ensured the reliability and specificity of experimental data. These techniques can be employed to effectively manage the potential impact of maternal age, underlying pathology, and glucose sensitivity on the experimental results, ensuring a more precise evaluation of the findings. To confirm the consistency of the experimental results, the analysis of the samples was repeated multiple.

Furthermore, fluctuating blood sugar levels and long-term inflammatory status can indeed have a significant impact on the metabolic level of HSCs. Therefore, in the early phase of the experimental design, a series of screening criteria were established for disease status and age of donors to ensure sample homogeneity and reliability. The blood levels of the primary inflammatory marker (C-reactive protein) were also measured. The sample donors were all disease-free and had normal blood glucose levels. The CB sources did not include any elderly or high-risk mothers. The donors received standardized nutritional support before delivery, and showed no evidence of inflammatory conditions or fluctuations in blood sugar levels. The establishment of this methodological foundation allows further investigation of changes in the metabolic profiles of HSCs under different physiological conditions. In previous research, lipopolysaccharide (LPS) has been used to simulate inflammatory responses in animal models. It was observed that LPS can induce a temporary increase in oxidative phosphorylation levels, leading to the temporary transition of HSCs from a quiescent state. The establishment of these methods can better enable us to observe changes in blood sugar and the impact of inflammatory status on HSC metabolism. Based on this methodological procedure, our next study will examine the impact of these differences on the differentiation pathways of HSCs and analyze the differences in metabolic levels of HSCs under various physiological states. This will allow an improved understanding of the functional and behavioral features of HSCs under different metabolic conditions.

Here, the potential value of analyzing metabolic functionality as a means of distinguishing among hematopoietic cells at different stages in the differentiation process was assessed. Hematopoietic differentiation was associated with a high degree of metabolic heterogeneity. Flow cytometry is capable of detecting all metabolic proteins of interest in the appropriate fluorescent channels with compensation to eradicate interference between fluorophores. This method was used for the concurrent analysis of 20 primary types of CB-derived hematopoietic cells from all stages of differentiation, such as stem progenitor cells, subsets of granulocyte, monocyte, B, and T cells. These cells were compared under identical experimental conditions using the same criteria, enabling analyses of anabolic and catabolic activities, as well as phosphate and glucose metabolism. This method thus allowed the development of a comprehensive metabolic map of hematopoietic cells during different phases of differentiation. Transcriptomic methods were also used to investigate the metabolic heterogeneity of CB-derived hematopoietic cells. These findings offer a solid methodological approach and reference values for conducting systematic analyses of cellular metabolic activity and directing future research on the association between metabolism and aging or cancer.

## Data Availability

The datasets presented in this study can be found in online repositories. The names of the repository/repositories and accession number(s) can be found in the article/**Supplementary Material**.
