# Supplementary material for: Met-Flow analyses of the metabolic heterogeneity associated with different stages of cord blood-derived hematopoietic cell differentiation
Source: Front Immunol. 2024 Oct 17;15:1425585. doi: 10.3389/fimmu.2024.1425585 (PMC11524850; doi:10.3389/fimmu.2024.1425585)
Supplement: Supplementary file 1 [file DataSheet1.pdf]

Supplementary Material

Supplementary Figure Captions

Figure S1. Flow cytometry plot of 20 populations of hematopoietic cell populations isolated from the human cord blood

Supplementary table captions

Table S1. Immunophenotypes of hematopoietic cells for flow cytometry

Table S2. Antibody combination

Table S3. The catalogs for Antibodies

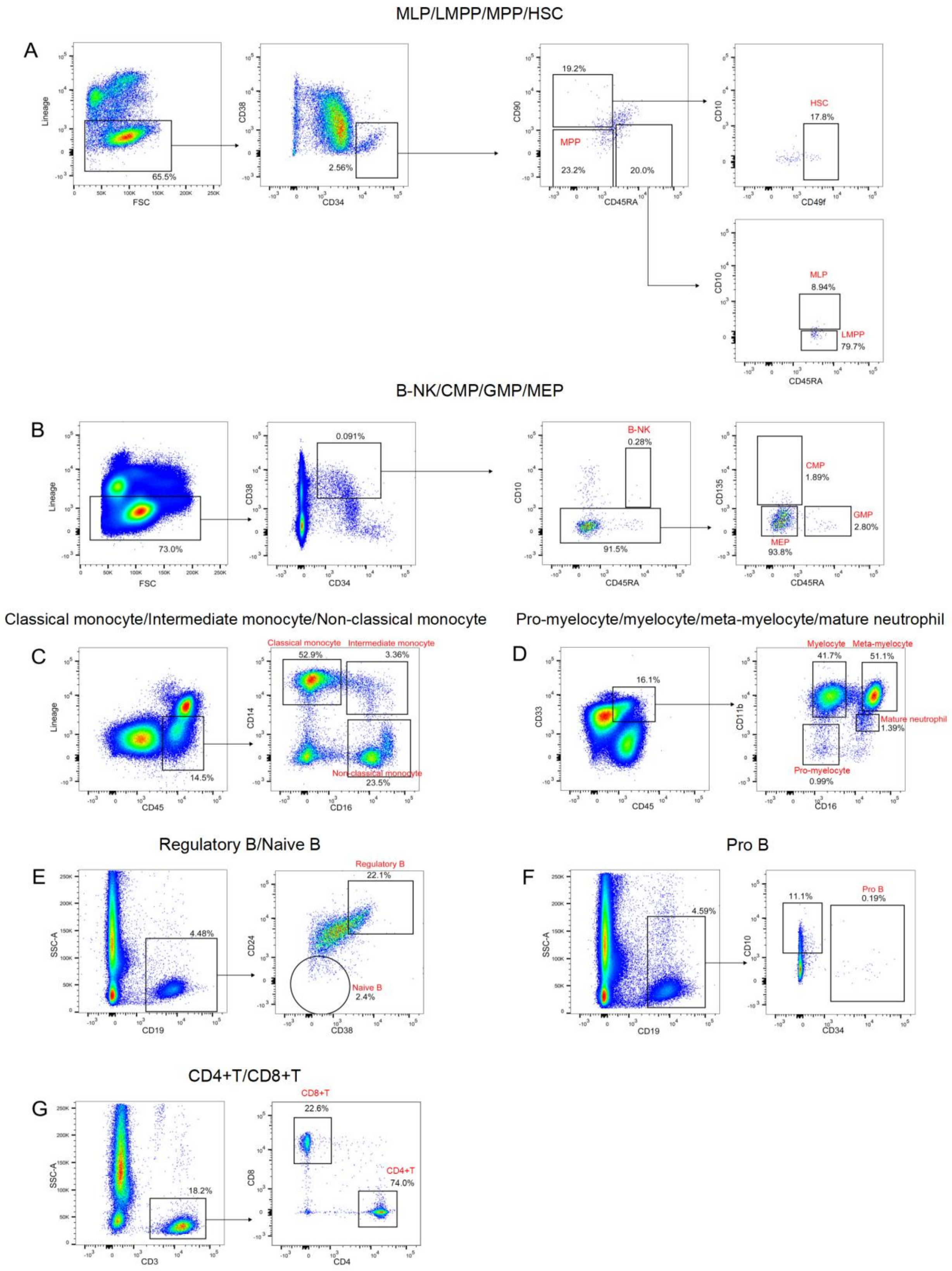
MLP/LMPP/MPP/HSC
A
Lineage
FSC
65.5%
CD38
CD34
2.56%
CD90
CD45RA
19.2%
MPP
23.2%
20.0%
CD10
CD49f
HSC
17.8%
MLP
8.94%
LMPP
79.7%
B-NK/CMP/GMP/MEP
B
73.0%
0.091%
B-NK
0.28%
91.5%
CD135
CMP
1.89%
GMP
2.80%
MEP
93.8%
Classical monocyte/Intermediate monocyte/Non-classical monocyte
C
CD45
14.5%
Classical monocyte
52.9%
Intermediate monocyte
3.36%
CD14
CD16
Non-classical monocyte
23.5%
Pro-myelocyte/myelocyte/meta-myelocyte/mature neutrophil
D
CD33
16.1%
CD11b
Myelocyte
41.7%
Meta-myelocyte
51.1%
Mature neutrophil
1.39%
Pro-myelocyte
0.99%
Regulatory B/Naive B
E
SSC-A
CD19
4.48%
Regulatory B
22.1%
CD24
Naive B
2.4%
Pro B
F
4.59%
11.1%
Pro B
0.19%
CD4+T/CD8+T
G
CD3
18.2%
CD8+T
22.6%
CD8
CD4
CD4+T
74.0%

Figure S1. Flow cytometry plot of 20 populations of hematopoietic cell populations isolated from the human cord blood. A. Hematopoietic stem cell (HSC) population, pluripotent progenitor cell (MPP) population, lympho-myeloid primed progenitor (LMPP) population and multipotent lymphoid progenitors (MLP) population; B. common myeloid progenitor (CMP) population, granulocyte-macrophage progenitor (GMP) population, megakaryocyte erythroid progenitor (MEP) population and B-NK progenitor cell (B-NK) population; C. classical monocyte, intermediate monocyte and non-classical monocyte populations; D. pro-myelocyte, myelocyte, meta-myelocyte and mature neutrophil populations. E. regulatory B and naive B cell populations. F. pro-B cell population. G. $CD4^+$ T and $CD8^+$ T cell populations.

Table S1. Immunophenotypes of hematopoietic cells for flow cytometry.

Immunophenotype	Surface marker
HSC	$Lin^{-}CD34^{+}CD38^{-}CD90^{+}CD45RA^{-}$
	$CD49f^{+}CD10^{-}$
MPP	$Lin^{-}CD34^{+}CD38^{-}CD90^{-}CD45RA^{-}$
MLP	$Lin^{-}CD34^{+}CD38^{-}CD90^{-}CD45RA^{+}CD10^{+}$
LMPP	$Lin^{-}CD34^{+}CD38^{-}CD90^{-}CD45RA^{+}CD10^{-}$
CMP	$Lin^{-}CD34^{+}CD38^{+}CD10^{-}CD135^{+}CD45RA^{-}$
GMP	$Lin^{-}CD34^{+}CD38^{+}CD10^{-}CD135^{+}CD45RA^{+}$
MEP	$Lin^{-}CD34^{+}CD38^{+}CD10^{-}CD135^{-}CD45RA^{-}$
B-NK	$Lin^{-}CD34^{+}CD38^{+}CD45RA^{+}CD10^{+}$
classical monocyte	$Lin^{-}CD45^{+}CD16^{-}CD14^{+}$
Intermediate monocyte	$Lin^{-}CD45^{+}CD16^{+}CD14^{+}$
non-classical monocyte	$Lin^{-}CD45^{+}CD16^{+}CD14^{-}$
CD4+T	$CD3^{+}CD4^{+}CD8^{-}$
CD8+T	$CD3^{+}CD4^{-}CD8^{+}$
pro-B	$CD19^{+}CD10^{+}CD34^{+}$
regulatory B	$CD19^{+}CD24^{+}CD38^{+}$
naive B	$CD19^{+}CD24^{-}CD38^{-}$
pro-myelocyte	$CD33^{+}CD45^{+}CD11b^{-}CD16^{-}$
myelocyte	$CD33^{+}CD45^{+}CD11b^{+}CD16^{-}$
meta-myelocyte	$CD33^{+}CD45^{+}CD11b^{+}CD16^{low}$
mature neutrophil	$CD33^{+}CD45^{+}CD11b^{+}CD16^{+}$

Lineage markers: CD3, CD14, CD16, CD19, CD20, CD56.

Table S2. Antibody combination.

Name of antibody combination	Antibodies
B-NK/CMP/GMP/MEP	FITC-Lineage markers, PE-Cy7 anti CD38, APC anti CD34, PercP-Cy5.5 anti CD90, APC-Cy7 anti CD45RA, PE anti CD135, BV510 anti CD10
MLP/LMPP/MPP/HSC	FITC-Lineage markers, PE-Cy7 anti CD38, APC anti CD34, PercP-Cy5.5 anti CD90, APC-Cy7 anti CD45RA, PE anti CD10, BV510 anti CD49f
classical monocyte/intermediate monocyte/non-classical monocyte	FITC-Lineage markers, PE-Cy7 anti CD45, APC anti CD16, PE-Cy7 anti CD14
pro-myelocyte/myelocyte/meta-myelocyte/mature neutrophil	PercP-Cy5.5 anti CD33, PE-Cy7 anti CD45, APC anti CD16, PE anti CD11b
$CD4^+$ T/$CD8^+$ T	BV510 anti CD3, PE anti CD4, APC anti CD8
regulatory B/naive B	PE-Cy7 anti CD19, PE anti CD24, APC anti CD38
pro-B	PE-Cy7 anti CD19, BV510 anti CD10, APC anti CD34

Table S3. The catalogs for Antibodies.

Antibody	Clone number	Fluorochrome	Catalog No.	Company
CD34	581	APC	555824	BD Biosciences
CD38	HB7	PE-Cy7	25-0388-42	Invitrogen
CD38	HIT2	APC	560980	BD Biosciences
CD10	HI10a	BV510	563032	BD Biosciences
CD10	HI10a	PE	312203	Biolegend
CD45RA	HI100	APC-Cy7	304127	Biolegend
CD135	4G8	PE	558996	BD Biosciences
CD90	5E10	Percp-Cy5.5	561557	BD Biosciences
CD49f	GoH3	BV510	563271	BD Biosciences
CD45	2D1	PE	368530	Biolegend
CD45	2D1	PE-Cy7	368531	Biolegend
CD14	63D3	PE-Cy7	367112	Biolegend
CD16	3G8	APC	302011	Biolegend
CD33	P67.6	Percp-Cy5.5	341650	BD Biosciences
CD11b	M1/70	PE	101207	Biolegend
CD3	OKT3	BV510	317332	Biolegend
CD4	SK3	PE	344605	Biolegend
CD8	SK1	APC	344721	Biolegend
CD19	HIB19	PE-Cy7	302215	Biolegend
CD24	ML5	PE	311105	Biolegend
ASS1	EPR12398	No	AB273048	Abcam
GLUT1	EPR3915	No	AB231684	Abcam
IDH2	EPR7577	No	AB131263	Abcam
ACAC	EPR23235-47	No	AB269272	Abcam
PRDX2	EPR5154	No	AB109367	Abcam
HK1	EPR10134(B)	No	AB150423	Abcam
ATP5A	EPR13030(B)	No	AB176569	Abcam
G6PD	EPR20668	No	AB210702	Abcam
CPT1A	EPR21843-71-2F	No	AB234111	Abcam
